# Reactivity of Different Crystalline Surfaces of C_3_S During Early Hydration by the Atomistic Approach

**DOI:** 10.3390/ma12091514

**Published:** 2019-05-09

**Authors:** K. M. Salah Uddin, Bernhard Middendorf

**Affiliations:** Department of Structural Materials and Construction Chemistry, University of Kassel, Mönchebergstraße 7, 34125 Kassel, Germany

**Keywords:** cement hydration, dissolution of C_3_S, alite, free energy surfaces, surface properties, molecular dynamics simulation, ReaxFF, metadynamics

## Abstract

Early hydration of tricalcium silicate (C_3_S) has received great attention over the years due to the increased use of composite cement with a reduced number of clinker phases, especially the addition of what should be very reactive C_3_S to guarantee early strength. Although many mechanisms have been proposed, the dissolution of polygonal C_3_S at the material interface is not yet fully understood. Over the last decade, computational methods have been developed to describe the reaction in the cementitious system. This paper proposes an atomistic insight into the early hydration and the dissolution mechanism of calcium from different crystalline planes of C_3_S using reactive force field (ReaxFF) combined with metadynamics (metaD). The reactivity and thermodynamic stability of different crystal planes were calculated from the dissolution profile of calcium during hydration at 298 K. The simulation results, clearly describe the higher reactivity of ($0\bar{1}\bar{1}$), (011), (100), and $\left(\bar{1}00\right)$ surfaces of C_3_S due to the strong interaction with the water, whereas, the dissolution profile explains the lower reactivity of ($\bar{1}\bar{1}0$), (110), ($0\bar{1}0$) and the effect of water tessellation on the (001), (010) planes.

## 1. Introduction

Concrete is the most used construction material on earth due to its sustainability and relatively low production cost. Cement is one of the main ingredients of concrete which is usually a solid mixture of different clinker phases [1,2]. However, the production process of cement clinkers not only has high energy consumption but also attributed to the release of 5% of global anthropogenic carbon dioxide (CO_2_) emissions [3]. Therefore, designing green cement with lower CO_2_ emissions and improved properties has become an important challenge in the construction sector. Despite continued research, the hydration mechanism of cement at the material interface remains a challenge. Tricalcium silicate (C_3_S, Ca_3_SiO_5_, alite) (Table A2) is the major constituent and the most reactive phase of the production of ordinary Portland cement. Moreover, C_3_S is the key source of the main hydration product named calcium silicate hydrates (C-S-H), which is responsible for the mechanical properties of concrete. Therefore, many numerical, as well as experimental studies, have been carried out to understand the hydration reaction by considering C_3_S as a model system [4]. Hydration mechanisms, especially early hydration at the atomistic scale, are not understood well due to the complexity of the process and a lack of experimental techniques. Experimental methods, i.e., X-ray diffraction, electron microscopy, nuclear magnetic resonance spectroscopy, and Infrared spectroscopy, do not sufficiently describe the interaction between C_3_S-water interface at the atomistic scale. However, molecular dynamics (MD) techniques are being used to investigate the nanoscale process in order to provide deeper insights into the problem. Additionally, most of the computer-based cement hydration processes have been simplified by considering the cement particle as a sphere [5,6,7]. In reality, the polygonal C_3_S particle consists of different crystal planes with different reactivity due to the atomic arrangement of the respective surfaces. They are unable to explain the reactivity of the individual surfaces toward hydration. Atomistic simulation using reactive force fields (ReaxFF) have been developed over the past decade to investigate the reaction at the material interface with sufficient accuracy. Various simulations have been executed successfully in order to explain the reactions of hydrocarbons [8] metal oxides (Si/SiO_2_) [9], metal hydrides [10], polymer chemistry [11] and many other systems. ReaxFF has allowed calculating molecular dynamics in femtosecond (10^−15^ seconds) time steps to get correct integration of equations of motion. However, sometimes it becomes computationally expensive during the calculation of transition state (TS), which occurs in a larger time scale (ns). In order to solve this timescale problem, metadynamics (metaD) is integrated into ReaxFF. Metadynamics is a powerful sampling method that can accelerate the calculation of the reaction path by introducing a biased potential and observing the rare events (TS) within a smaller time scale [12]. Hence, a combination of ReaxFF and metaD has shown great potential to investigate the reaction mechanism with sufficient accuracy. Hydration mechanism involved dissolution, the reaction in a pore solution (homogeneous nucleation) and precipitation. Manjano et al. explained the absorption of water on the C_3_S surfaces successfully by using ReaxFF [13]. Unfortunately, the authors did not study the reaction pathways for the dissolution of calcium. In this paper, multistep approaches are taken to explain the reactivity of different surfaces of C_3_S at 298K. Initially, the different crystalline planes of C_3_S were hydrated for 600 picoseconds using ReaxFF (Table A1). Afterward, pre-hydrated surfaces were used to study the reactivity from the dissolution profile of calcium (free energy surfaces).

## 2. Methods and Modeling Approach

ReaxFF is able to describe both bond formation and bond breaking by calculating the bond order of each atom pair. The evolution of bond order modifies the other connectivity term (i.e., bond angle, torsion) during MD simulation. The non-bonded interactions (i.e., electrostatic, van der Walls) are also calculated for all atom pairs. More details about the ReaxFF can be found in the references [8,14]. In addition, ReaxFF has been implemented in the cementitious systems using combined parameter sets, Si-O-H and Ca-O-H, developed independently by Fogarty et al. and Manzano et al., respectively [9,15]. This parameter set yields excellent results for the investigation of mechanical properties of amorphous, crystalline C-S-H and the hydration mechanism of C_3_S [13]. The combined parameter set is used for all MD simulation presented in this paper. Furthermore, metaD is coupled with ReaxFF to reduce simulation cost for calculating the TS of a reaction. Metadynamics is one of the powerful algorithms that can accelerate the observation of rare events by introducing a biased potential that acts on a selected number of degrees of freedom named as collective variables (CVs) [16]. MetaD is able to enhance the sampling by reconstructing the free energy surfaces (FES). The bias potential is applied as a sum of Gaussians, continuously growing during MD simulation by acting directly on the microscopic coordinates of the system [17]. The simulations were carried out by ReaxFF implemented in the latest version of LAMMPS (Large-scale Atomic/Molecular Massively Parallel Simulator, Stable version (17 Feb. 2018), developed jointly by Sandia National Laboratories, New Mexico, and Temple University, Philadelphia, USA) simulation packages [18]. Additionally, the metaD simulations were performed by using the PLUMED package as an extension of LAMMPS [19].

### Model Construction

The fresh cleaved (011) C_3_S orthogonal periodic simulation cell (32.77 × 47.71 × 27.81) × 10^−30^ m^3^ composed of 3165 atoms was constructed by virtual nano lab (VNL) and Avogadro [20,21,22]. The geometry was optimized using energy minimizations with Hessian-free truncated Newton algorithm (hftn) where the cutoff tolerances for energy and force were 4.18 × 10^−4^ and 4.18 × 10^−8^ kJ mol^−1^ respectively. Maximum iterations for minimizer were 100. Later on, an additional 3.13 × 10^−26^ m^3^ periodic cell (20 Å on top of the surface) filled with water was added to the optimized (011) surface of C_3_S using packmole [23]. The number of water molecules was 695 and matched a density of 1000 kg m^−3^ with a random distribution. The simulation cells with a total of 5250 atoms were equilibrated to 298K and 1 atm for 150 picoseconds with 0.5 femtoseconds time steps using canonical ensemble with a Nose-Hoover thermostat (nvt), integrating the non-Hamiltonian equations of motion. Subsequently, they were hydrated for 600 picoseconds using isothermal-isobaric (npt) ensembles with all three diagonal components of the pressure tensor coupled together (iso) [24,25]. A periodic boundary condition was applied during the simulation.

The last geometry of (011) hydrated surfaces of C_3_S was taken (after 600 picoseconds) to calculate the dissolution mechanism of calcium using the ReaxFF coupled with metaD. The PLUMED package was used as an extension of LAMMPS to perform the metaD simulation [17]. It enforced the reaction using history dependent bias potential. The calcium atom (Ca-3013) from pre-hydrated (011) surface of C_3_S positioned in between two silicates was selected (Table 1) to be removed from the surface-to-pore solution, applying well-tempered metaD. The distance (collective variable) between the center of mass (COM) and the selected calcium atom was computed by adding biased potential as a Gaussian with frequency 40. Furthermore, Gaussian hills with a height of 6.28 kJ /mol and a full width at half-maximum of 0.2 × 10^−10^ m were added every 0.02 picoseconds. The metaD coupled with ReaxFF was performed for 150 picoseconds (till converged) using the npt ensemble at temperature 298K and the energies were averaged over the entire period to compute the free energy of dissolution. The dissolution pathways (free energy surface) of calcium were computed for (011) surfaces of C_3_S and compared the surface reactivity by analyzing the activation energy and free energy change (ΔG) during dissolution. A similar approach was used for other cleavage planes (Table A1) to simulate hydration. The reactivities were calculated from the dissolution profile (FES) of calcium of the particular surface. 

## 3. Results and Discussion

### 3.1. Hydration of C_3_S

**Figure 1:**
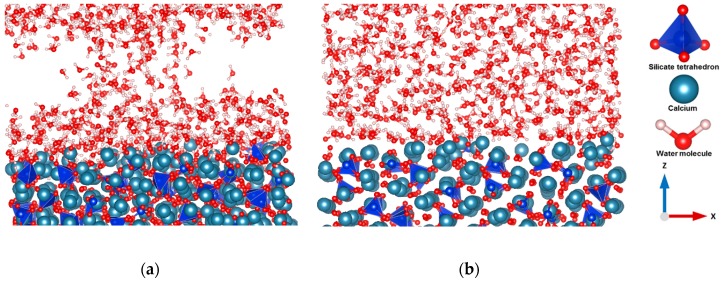
Comparison of the reactivity between the ($0\bar{1}\bar{1}$) surface and (001) (**a**,**b**) during hydration for 600 picoseconds at 298 K.

To understand the interaction between the different crystal planes of C_3_S and bulk water, the reaction dynamics over the period of 600 picoseconds at room temperature and pressure were followed. In addition, a periodic boundary condition was applied to solve the atom loss problem during the simulation. The time step of the entire simulation was 0.5 fs to track the movement of lighter hydrogen present in the simulation system. The hydration was limited to 600 picoseconds in order to avoid polymerization of silicate. Using the hydrated surfaces as a starting geometry, the surface polymerization was studied. It was observed that initially, the crystal surfaces were unstable due to the undercoordinated atoms and broken bonds. However, as time went on, water molecules interacted with the surfaces of C_3_S and dissociated from hydroxide by protonating the free oxygen and oxygen of silicate monomer, which leads to the minimization of the surface energy. Later on, proton transfer occurred by the hopping process. A strong interaction between water and the (100), (101), (011), ($0\bar{1}\bar{1}$) plane of C_3_S was observed. Free oxygens of these surfaces are one of the influential factors for increasing reactivity. Water molecules shifted toward the surfaces in both (011), ($0\bar{1}\bar{1}$) planes (periodic boundary condition) due to the strong interaction. As a result, an air void was observed which affected the formation morphology of capillary pores during hydration. The hydration was limited to 600 picoseconds and the transport mechanism of water was not intended to be observed. (Figure S1). In contrast, the (001), (010), ($0\bar{1}0$), (110), ($\bar{1}\bar{1}0$) crystal surfaces showed less reactivity during hydration. Water tessellation was observed on the (001) surfaces of C_3_S, which stabilized the surface (Figure 1b). These results correspond with Manzano et al. [13]. Further study was undertaken to get a clear overview of the different reactivity of different cleavage planes of C_3_S. The dissolution profile of the selected calcium located on the different surfaces using metaD coupled with ReaxFF provided an exact explanation about the reactivity by comparing the total free energy changes during dissolution. 

### 3.2. Dissolution of Calcium from C_3_S

**Figure 2:**
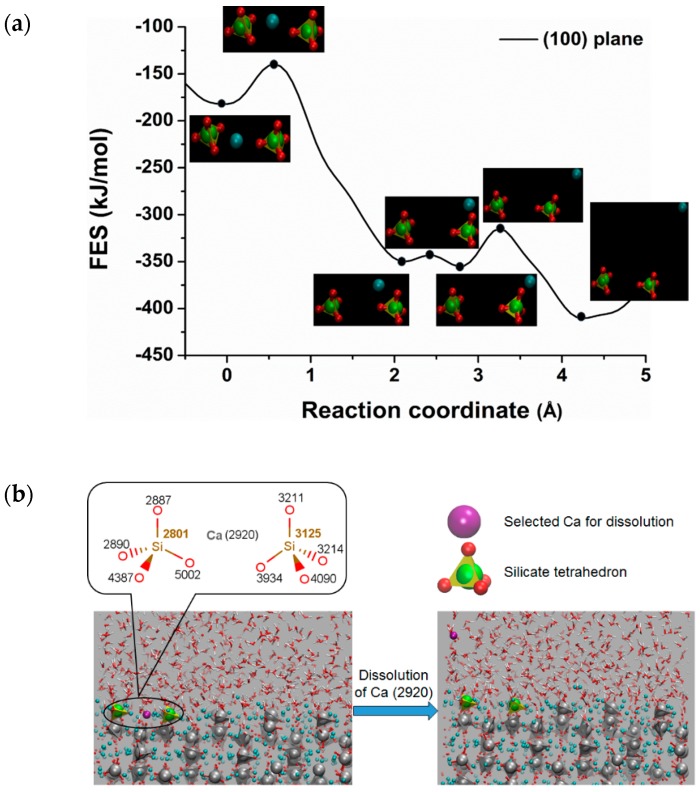
(**a**) Dissolution profile (free energy surface) of Ca2920 from (100) surface of C_3_S at RT (298K) (distance ×10^−10^) m (**b**) Snapshot of calcium dissolution process from the surface to pore solution.

**Figure 3:**
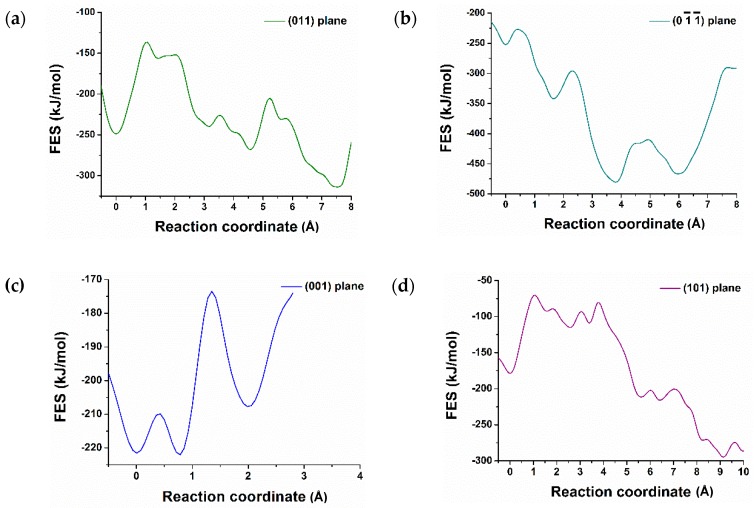

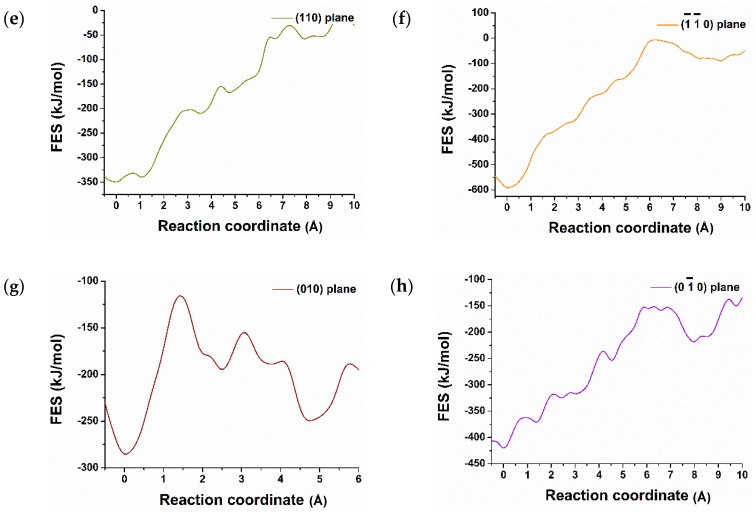
Dissolution profile (free energy surface) of calcium from (011), ($0\bar{1}\bar{1}$), (001), (101), (110), ($\bar{1}\bar{1}0$), (010), ($0\bar{1}0$) surfaces of C_3_S (**a**–**h**) and reactivity difference among them at 298 K.

Free energy calculation has received great attention in molecular dynamics simulation in order to get a clear impression of reaction pathways including transition state. Well-tempered metaD is an excellent method that enforces the reaction to overcome the activation barrier by selecting the proper CVs. Selecting the appropriate CVs is a challenging task in the metaD simulation. The main advantage of metaD is to control and compute the FES of the targeted region. In this study, the metaD coupled with ReaxFF to calculate the dissolution profile of calcium from the surfaces of C_3_S and to compute the free energy surfaces (FES) of the region was applied. Figure 2a represents the free energy profile for the dissolution of calcium from (100) surface of C_3_S at 298K using the single collective variable distance between the Ca-2920 and center of the mass of the crystal. The X-axis represents the reaction coordinate in terms of distance in Å (10^−10^ m), starting from the initial state (on the surface) at zero to the final state (in the solution) at the next lowest minima. The calcium located between two silicates has an influential factor to prevent the dimerization of silicate. This specific type of calcium for dissolution was purposely targeted to study the surface polymerization in the future. The free energy profile calculated from the metaD run represents the movement of Ca-2920 from between the two silicates to the pore solution by overcoming the first barrier of 46.00 kJ/mol at 0.60 × 10^−10^ m. After a small fluctuation, it overcomes the electrostatic interaction with the oxygen of silicate and dissolute completely into the pore solution by passing the energy barrier of 37.60 kJ/mol at 3.30 × 10^−10^ m (Figure 2b). The total free energy change (*ΔG*) of −225.90 kJ/mol and lower activation barrier were directed to the exergonic (thermodynamically favorable process) and highly reactive surface respectively. Although the polymerization of silicate was not intended to be studied here, it was observed that the polymerization was not possible due to the strong electrostatic interaction of the surrounding calcium. Therefore, a detailed study is required to investigate the critical condition for the surface polymerization. Similarly, (101), (011), ($0\bar{1}\bar{1}$) surfaces of C_3_S are found as reactive toward hydration. Among them, ($0\bar{1}\bar{1}$) was shown to have the maximum reactivity (*ΔG* = −214.20 kJ/mol) which explains the air void formation due to the strong interaction, i.e., movement of water to the surface (Figure 3b).

Contrariwise, the dissolution profile of calcium from (001) surface shows the opposite trend and supports the results that were predicted after 600 picoseconds of hydration (Figure 1b). The complete dissolution of Ca-1957 from (001) surface was required to overcome the barrier of 12.50 kJ/mol at 0.43 × 10^−10^ m and 50.00 kJ/mol at 20 × 10^−10^ m. The total free energy change (ΔG) of +14.00 kJ/mol at 298 K represents an endergonic reaction and is thermodynamically unfavorable [26,27]. This explains the reduced reactivity and water tessellation of the (001). Likewise, Ca dissolution from (010), ($0\bar{1}0$), (110), ($\bar{1}\bar{1}0$) surfaces required a very high activation barrier (Table 1), therefore, these surfaces are less reactive toward hydration, endergonic and thermodynamically not favorable. 

## 4. Conclusions

This paper explores the potential of ReaxFF coupled with the metaD modeling approach to understand the early hydration of C_3_S as well as to observe the reactivity of different crystalline planes from the dissolution mechanism of calcium. The dissolution profile results can be summarized as (100), (101), (011) and ($0\bar{1}\bar{1}$) surfaces being reactive and thermodynamically favorable toward hydration at 298 K. They also have shown a clear indication of air void formation as a result of their strong interaction with water. Moreover, it was observed that water tessellation was a reason for lower reactivity of (001), ($0\bar{1}0$) and ($\bar{1}\bar{1}0$) surfaces of C_3_S during hydration. Among all the cleavage planes of C_3_S, ($0\bar{1}\bar{1}$) was found to be the most reactive and ($\bar{1}\bar{1}0$) the least reactive.

## Figures and Tables

**Table 1:** Free energy change of different surfaces of C_3_S during the dissolution of calcium.

Crystal Plane of C_3_S	The Atomic ID of the Selected Ca for Dissolution	Free Energy of Activation $\left(\Delta G^{*}\right)$ kJ/mol	Free Energy Change (ΔG) kJ/mol	Thermodynamic Properties
($0\bar{1}\bar{1}$)	2994	25.60	−214.20	Exergonic
(100)	2920	46.00	−225.90	Exergonic
(011)	3013	112.80	−65.00	Exergonic
(101)	3048	108.40	−116.10	Exergonic
(001)	1957	50.00	+14.00	Endergonic
(010)	2471	169.70	+36.00	Endergonic
($0\bar{1}0$)	2352	267.10	+202.10	Endergonic
(110)	3043	319.10	+291.50	Endergonic
($\bar{1}\bar{1}0$)	2998	584.70	+502.00	Endergonic

## Supplementary Materials

The following are available online at https://www.mdpi.com/1996-1944/12/9/1514/s1, Figure S1. Representing snapshot of (100), (101), (011), ($0\bar{1}\bar{1}$), (001), (010), ($0\bar{1}0$), (110), ($\bar{1}\bar{1}0$), surfaces of C_3_S (a–i) after hydration for 600 picoseconds at 298 K.

## Appendix A

**Table A1:** Crystallographic data for the orthogonal simulation cells of different planes of C_3_S.

Crystal Plane of C_3_S	Orthogonal Cell Dimension (Å^3^ / 10^-30^ m3)	No of Atoms in the Simulation Cell
(100)	56.49, 34.39, 37.06	5799
(101)	32.96, 34.39, 59.06	6003
(001)	28.37, 50.45, 49.91	5514
(010)	32.28, 37.78, 47.66	5115
($0\bar{1}0$)	32.77, 38.35, 48.37	5115
(110)	38.35, 43.35, 46.45	6538
($\bar{1}\bar{1}0$)	38.35, 43.35, 46.45	6536
(011)	32.77, 47.71, 47.81	5250
($0\bar{1}\bar{1}$)	32.77, 47.71, 47.81	5248

**Table A2:** List of abbreviations.

MD	Molecular Dynamics
C_3_S	Tricalcium silicate (Ca_3_SiO_5,_ alite)
C-S-H	Calcium Silicate Hydrates
ReaxFF	Reactive Force Field
LAMMPS	Large-scale Atomic/Molecular Massively Parallel Simulator
metaD	Metadynamics
TS	Transition State
CVs	Collective Variables
FES	Free Energy Surfaces
VNL	Virtual Nano Lab
hftn	Hessian-free truncated Newton algorithm
nvt	Nose-Hoover thermostat
npt	Nose-Hoover pressure barostat
