# Supplementary material for: Reactivity of Different Crystalline Surfaces of C3S During Early Hydration by the Atomistic Approach
Source: Materials (Basel). 2019 May 9;12(9):1514. doi: 10.3390/ma12091514 (PMC6539094; doi:10.3390/ma12091514)
Supplement: Supplementary file 1 [file materials-12-01514-s001.pdf]

materials

Reactivity of different crystalline surfaces of C_3S during early hydration by the atomistic approach

K. M. Salah Uddin [1,]* and Bernhard Middendorf [1,]*

Supplementary Materials

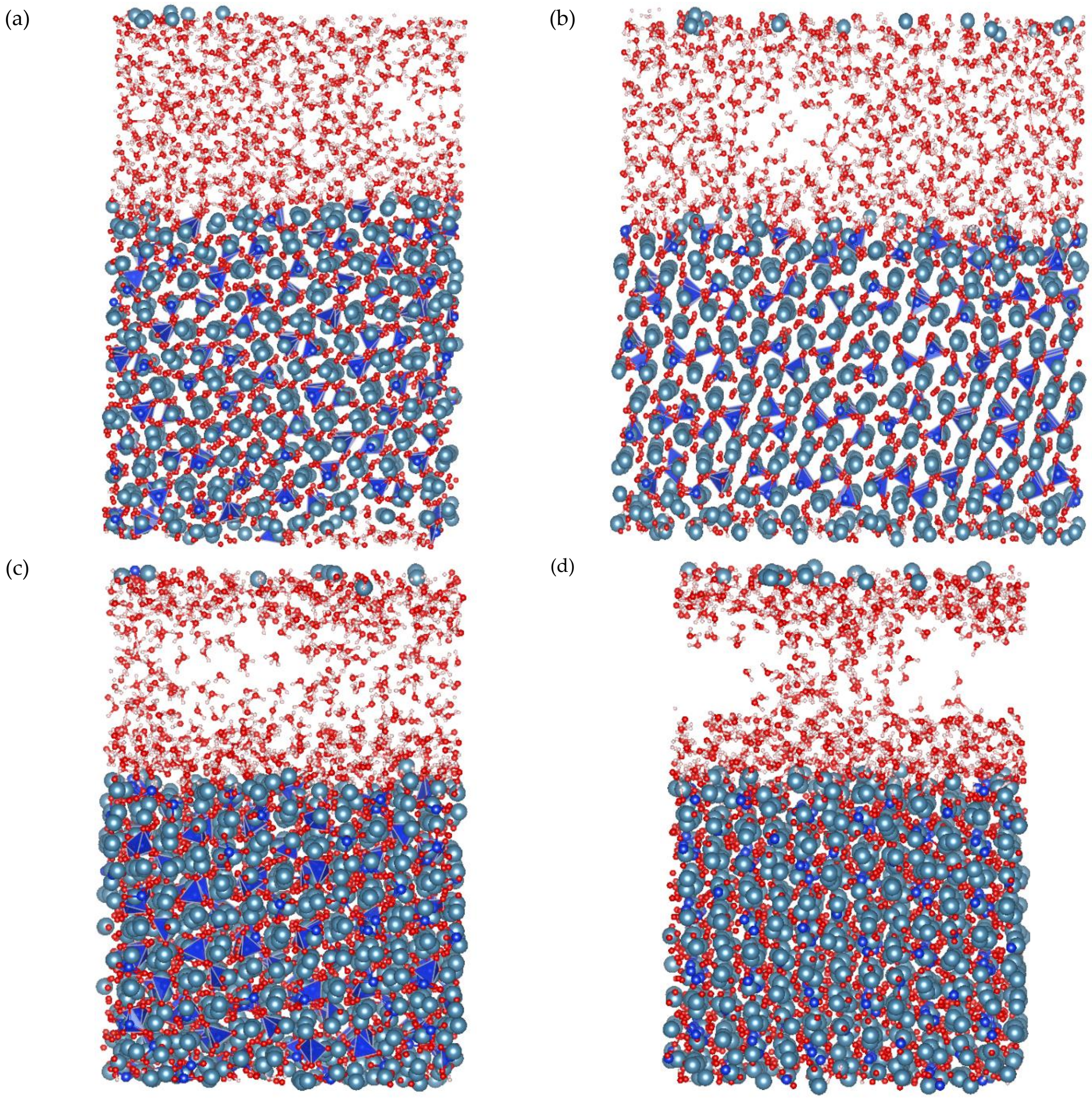

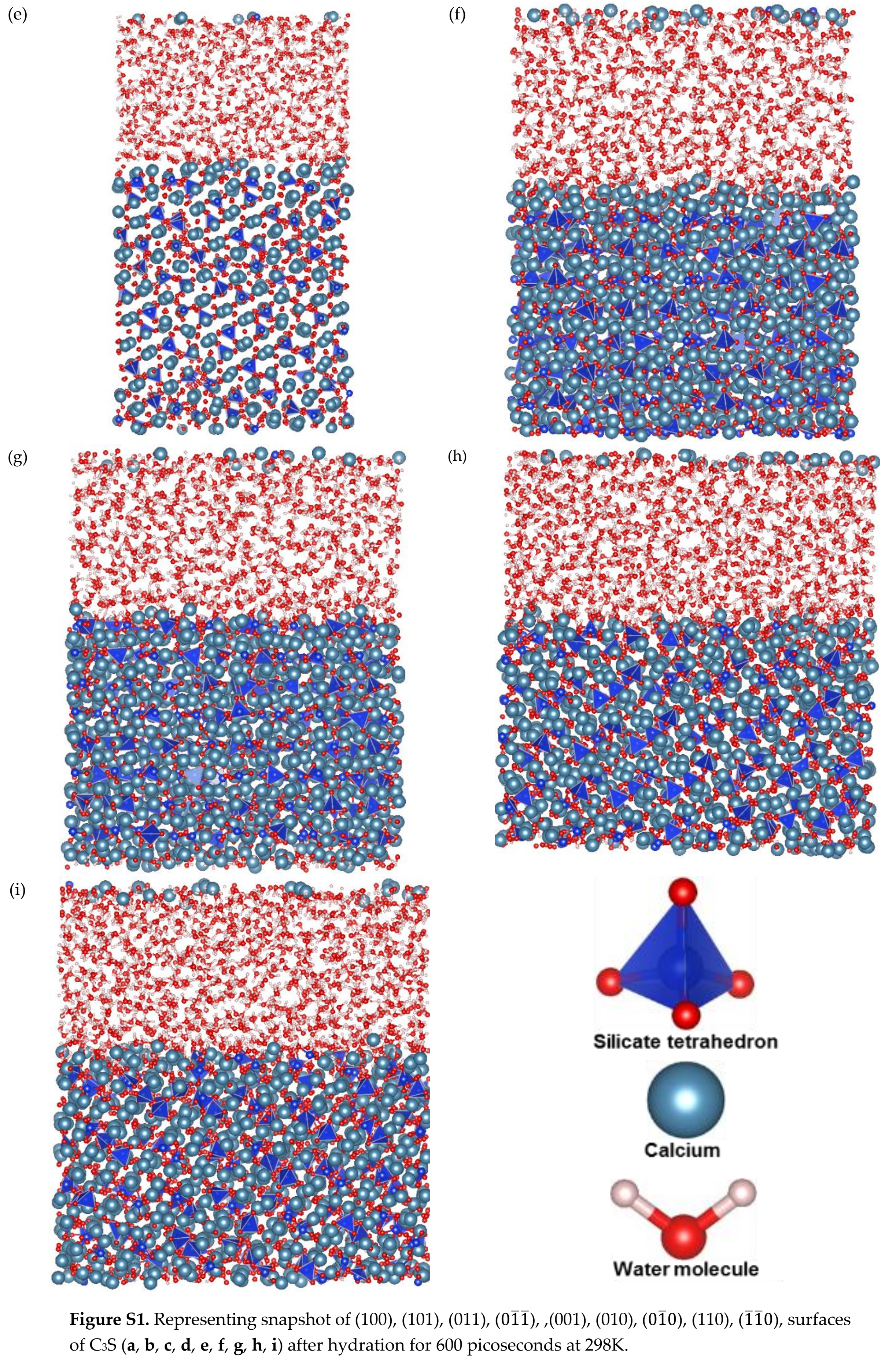

Figure S1. Representing snapshot of (100), (101), (011), (0$\bar{1}\bar{1}$), ,(001), (010), (0$\bar{1}$0), (110), ($\bar{1}\bar{1}$0), surfaces of C_3S (**a**, **b**, **c**, **d**, **e**, **f**, **g**, **h**, **i**) after hydration for 600 picoseconds at 298K.
